# Loss of Function of Endothelin-2 Leads to Reduced Ovulation and CL Formation

**DOI:** 10.1371/journal.pone.0096115

**Published:** 2014-04-24

**Authors:** Joseph A. Cacioppo, Sang Wook Oh, Hey-young Kim, Jongki Cho, Po-Ching Patrick Lin, Masashi Yanagisawa, CheMyong Ko

**Affiliations:** 1 Comparative Biosciences, College of Veterinary Medicine, University of Illinois, Urbana-Champaign, Illinois, United States of America; 2 Department of Biology Education, Institute of Fusion Science, Chonbuk National University, Jeonju, South Korea; 3 College of Veterinary Medicine, Research Institute of Veterinary Medicine, Chungnam National University, Daejon, South Korea; 4 Howard Hughes Medical Institute and Departments of Molecular Genetics, University of Texas Southwestern Medical Center, Dallas, Texas, United States of America; Michigan State University, United States of America

## Abstract

Endothelin-2 (EDN2), a potent vasoconstrictive peptide, is transiently produced by periovulatory follicles at the time of ovulation when corpus luteum (CL) formation begins. EDN2 induces contraction of ovarian smooth muscles *ex vivo* via an endothelin receptor A-mediated pathway. In this study, we aimed to determine if EDN2 is required for normal ovulation and subsequent CL formation *in?vivo*. In the ovaries of a mouse model that globally lacks the *Edn2* gene (*Edn2* knockout mouse; Edn2KO), histology showed that post-pubertal Edn2KO mice possess follicles of all developmental stages, but no corpora lutea. When exogenous gonadotropins were injected to induce super-ovulation, Edn2KO mice exhibited significantly impaired ovulation and CL formation compared to control littermates. Edn2KO ovaries that did ovulate in response to gonadotropins did not contain histologically and functionally identifiable CL. Intra-ovarian injection of EDN2 peptide results suggest partial induction of ovulation in Edn2KO mice. Endothelin receptor antagonism in wild type mice similarly disrupted ovulation, CL formation, and progesterone secretion. Overall, this study suggests that EDN2 is necessary for normal ovulation and CL formation.

## Introduction

Ovulation, a rupture of the ovarian surface and expulsion of the oocyte and cumulus cells from a mature follicle, is a central event in female reproduction that results in the formation of the corpus luteum (CL). Cross talk between circulating hormones and local cellular components in the hypothalamic-anterior pituitary-ovarian (HPO) axis is required to regulate oocyte production and rupture during ovulation [1], [2]. In humans, failure to ovulate, form CL, or produce progesterone are common causes of infertility and reproductive disorders [3], [4], and may arise from extrinsic or intrinsic ovarian complications. To further understand the intra-ovarian molecular mechanisms that ultimately allow ovulation to proceed, a multitude of studies over the past two decades have characterized gene product expression and actions relative to the reproductive cycle [2], [5], [6]. Of great interest are the molecules that are highly induced in the ovary only at the time of ovulation. Upon ovulation, the ruptured follicle transforms into a CL, the primary source of progesterone that is required for embryo implantation and pregnancy maintenance [7]. The follicular transformation to a CL is accompanied by two prominent changes: angiogenesis and cellular differentiation of granulosa and theca cells to luteal cells [8], [9]. A highly condensed vascular capillary network is established in the forming CL within hours of ovulation [10]. This newly formed luteal vasculature functions as a route for secreting progesterone and also for supplying nutrients, oxygen, and steroid hormone precursors to the CL. As CL formation is successive with ovulation and CL-secreted hormones are necessary for pregnancy, molecular mechanisms involved in CL formation and function are of interest in infertility therapy and contraceptive development.

Endothelin-2 (EDN2, bioactive peptide product of *Edn2* gene, seen also as ET-2 and previously as Vasoactive Intestinal Contractor/VIC) is present in both rodent and human ovaries [11], [12] and is transiently expressed in the granulosa cells immediately prior to ovulatory follicle rupture [13] via a hypoxia-driven pathway [14]. EDN2 is the least well studied member of the Endothelin system, which consists of three isoforms of 21-residue peptides (EDN1, EDN2, EDN3), two G-protein coupled receptors (ET_A_ and ET_B_), and two endothelin-converting enzymes (ECE-1 and ECE-2) [15], [16]. Little is known about what factors other than hypoxia are involved in *Edn2* induction [14], [17], [18]. EDN2 is best characterized as a vasoconstrictor [19]–[21], and triggers a contractile response when treated to ovarian tissue strips. Addition of tezosentan, a dual endothelin receptor antagonist drug, or BQ123, an ET_A_-specific antagonist, abolishes this effect of EDN2 [13]. Importantly, systemic administration of tezosentan prior to ovulation inhibits follicle rupture in superovulation-induced rats and mice [13], [22]. It has been previously proposed that follicular or ovarian contraction, which EDN2 may induce, serves as an ovulatory trigger for a follicle to rupture [23]. This idea is given credence by the presence of a smooth muscle network in the theca-interstitial layer of the ovary. However, no studies have conclusively demonstrated that ovarian contraction is necessary for ovulation.

Furthermore, EDN2 has recently been implicated in angiogenesis, cell migration, cell proliferation, and endothelial activation [11], [12], which are all necessary processes for CL formation. The CL is extremely vascular, and formation is dependent upon leukocyte invasion to direct tissue remodeling and rapid vessel growth through endothelial propagation. EDN2 has highest expression in the gastrointestinal tract [24], [25] where it may modulate immune function [26]. It is also seen in the photoreceptors of the eyes [27]–[29], where overexpression of EDN2 has been shown to favor the formation of tip cells in the developing vascular endothelium, which are necessary for angiogenesis [30]. EDN2 may also act as a survival factor in certain cancers [30]–[33] as well as a chemoattractant for macrophages [34]. Recent studies show that Endothelin induces VEGF secretion in a variety of cell types including ECs, smooth muscle cells, and cancer cells [35]–[38]. Klipper et?al. also showed that EDN2 directly induces VEGF in granulosa cells of the bovine ovary [17], and VEGF has also been shown to be necessary for CL formation [39], [40].

As increased EDN2 expression, follicle rupture, and initiation of CL formation are significant ovarian events that occur almost simultaneously, these findings led us to postulate that EDN2 both induces periovulatory follicular rupture and is necessary for CL formation. The aims of this study were to investigate the physiological significance of EDN2 in ovulation and CL formation with (1) a global *Edn2* gene-deficient mouse model (Edn2KO) [41] and (2) through pharmacological antagonization of endothelin receptors in wild type (WT) mice. Edn2KO mice have been recently characterized by Chang et?al. (2013) as having emphysema, hypothermia, and decreased growth [41], but were chosen based on their availability as a model completely lacking EDN2. Both Edn2KO mice and mice receiving drug antagonization exhibited CL formation defects as well as significantly reduced ovulation. Our results extend previous findings by revealing novel roles of EDN2 in CL formation as well as confirming its role in ovulation.

## Materials and Methods

### Ethics Statement

This study was carried out in tight accordance with the recommendations in the Guide for the Care and Use of Laboratory Animals of the National Institutes of Health. This protocol was approved by the University of Kentucky Animal Care and Use Committee (Protocol: 1111M2006), and all efforts were made to minimize animal suffering.

### Reagents

Antibodies against CD31 (PECAM-1) were purchased from Santa Cruz Biotechnology Inc. (sc-1506; Dallas, TX); antibodies against Caspase-3 (Casp-3) from Cell Signaling Technologies (D175 5A1E; Beverly, MA); antibodies against α-Smooth Muscle Actin (αSMA) and cholesterol side chain cleavage enzyme (P450scc) were purchased from Abcam Inc. (ab5694-100, Cambridge, UK) and Chemicon Inc. (AB1294, Billerica, MA), respectively. CD31 produces a membranal stain, while Casp-3, αSMA, and P450scc produce cytoplasmic stains. Biotin-labeled goat anti-rabbit IgG was purchased from Vector Lab (BA-1000, Burlingame, CA) and streptavidin-horseradish peroxidase (HRP, 550946) conjugate from BD Biosciences (San Jose, CA). Pregnant Mare Serum’s Gonadotropin (PMSG) and human chorionic gonadotropin (hCG) were purchased from Sigma Chemical Co. (St. Louis, MO). EDN2 peptide was purchased from American Peptide Co., Inc. (Sunnyvale, CA). Other common molecular reagents were purchased from Invitrogen Life Technologies, Inc (Carlsbad, CA).

### Animals

**Figure 1:**
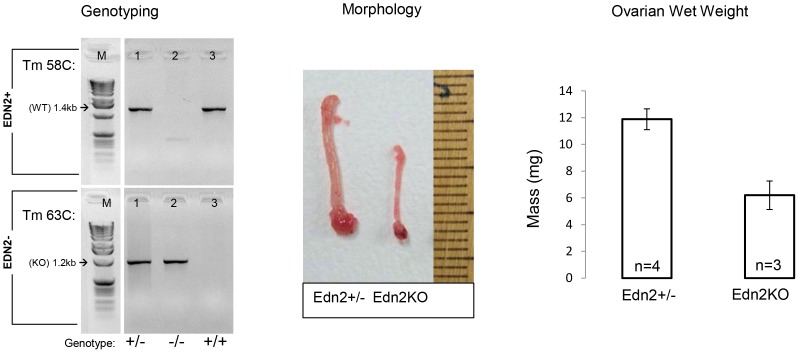
Genotyping, ovarian morphology and weight. Genotyping was performed as above using two reactions to distinguish between heterozygotes and Edn2KO mice. Lanes 1, 2, and 3 show Edn2+/?, Edn2KO, and WT mice that were genotyped from a single litter, respectively. Ovaries were collected from 23 to 31 day-old Edn2KO (n = 4) and age-matched Edn2+/? mice (n = 3) at the time of euthanasia 24 hours after superovulation. Note that Edn2KO ovaries and uteri were smaller than heterozygote siblings, and Edn2KO ovaries were also lighter when ovarian masses from mice aged 23–31 days were averaged (wet weight, p = 0.030). When average ovarian weight was normalized to average body weight, no significant difference remained between groups (p = 0.318).

All mice used were of the C57BL/6 genetic background and were maintained under controlled lighting (14 hour light/10 hour dark) with continuous access to food and water. Mouse genotypes were determined by PCR using three primers: 5?-AGG TGA CAC AAA ATA TTC TGT TCA GTC CAC-3?, 5?-GAG GAT TGG GAA GAC AAT AGC AGG CAT GC-3?, and 5?-GCA GAA GTA CAC ACA TTC CTT GTC-3? (Fig. 1). As their lifespan was expandable if they were kept warm, the Edn2KO mice and control mice were kept on a 30°C warm pad (Lectro-Kennel Heated Pet Met Model #1019, K&H Manufacturing, LLC.) from weaning to the end of the experiment. Puberty was assessed by the presence of a vaginal opening in female mice, which normally occurs around 5 weeks of age (32.4±3.1 days) in wild type mice in our colony. Post-pubertal female mice that demonstrated vaginal opening were sacrificed by CO_2_ asphyxiation and cervical luxation; their ovaries were collected, massed, and fixed in 4% paraformaldehyde. Tissues were imaged under a dissecting scope with a Leica DFC320 camera and then fixed in 4% paraformaldehyde for histology. To correlate magnification to actual size, images at 4X, 10X, 20X, and 40X have heights of 2389, 955, 478, and 239 microns and widths of 3241, 1297, 648, and 324 microns, respectively.

### Superovulation Treatment

Female pre-pubertal Edn2KO mice and controls were injected in the intra-peritoneal cavity with 5 IU of PMSG at the ages of 23–25 days to induce follicle development, and then 48 h later with 5 IU of hCG to induce ovulation, according to the gonadotropin primed model [13]. Animals were euthanized by CO_2_ asphyxiation and cervical luxation at 16 or 24 hours after hCG injection, blood was drawn by cardiac puncture, and reproductive organs were harvested. Oocytes were collected from the ampullas of each oviduct, exposed to 80 U/mL hyaluronidase (Sage Media, Trumbull, CT) and imaged. Sera were collected from whole blood using BD Microtainer Serum Separator Tubes (Franklin Lakes, NJ) and frozen at −20°C until analysis. Tissues were either frozen on dry ice and stored at −80°C or massed and fixed in 4% paraformaldehyde for histology.

### EDN2 Supplementation Treatment

Female prepubertal Edn2KO mice and control mice were superovulated at 23–25 days of age as above. Eleven hours after hCG injection, mice were placed under general anesthesia with isoflurane. Under sterile conditions, ovaries were individually surgically exteriorized. Right ovaries were injected with 10 ul of phosphate buffered saline (PBS) into the center of the ovary whereas left ovaries received either 10 ul of 10nM EDN2 (20 pmoles in PBS) or were un-injected. Ovaries were replaced into the body cavity. Mice were killed at 16 hrs after hCG. Oocytes and reproductive tissues were collected as above. Tissues were collected and fixed in 4% paraformaldehyde or frozen on dry ice.

### Pharmacological Inhibition of Endothelin Receptors in WT Mice

Female WT mice of 25 days of age were induced for superovulation as described above and received intraperitoneal (i.p.) injections of either 100 ul PBS or 100 ul of 50nM of the dual receptor antagonist drug tezosentan (Actelion Inc., South San Francisco, CA). To account for the half-life of tezosentan and maintain receptor antagonization, mice received i.p. injections of 100 ul every two hours from hCG 12– hCG 22 hours and were sacrificed at hCG 24 hours. Oocytes, reproductive tissues, and blood were collected as above. Ovaries were frozen on dry ice for cryo-sectioning.

### Histology and Immunohistochemistry

Ovaries fixed in 4% paraformaldehyde were embedded in paraffin blocks. For histology, blocks were cut at 7 um in serial sections by microtome, mounted on slides, and stained with hematoxylin/eosin. Immunohistochemistry was performed as described previously [13]; briefly, sections were de-paraffinized by treatment with xylenes and rehydrated through a graded alcohol series. Antigen unmasking was carried out by boiling the sections in sodium citrate buffer (10 mM sodium citrate; and 0.05% Tween 20, pH 6.0) for 10 min in a microwave oven. After being allowed to cool to room temperature, the sections were rinsed briefly in PBS and treated with 0.3% hydrogen peroxide (Sigma) in water for 30 min to quench endogenous peroxidase activity. Sections were incubated with 3% bovine albumin serum in 0.01 PBS to block non-specific binding for 20 min at room temperature. Sections were then incubated with anti-CD31, anti-Caspase-3 anti-αSMA, or anti-P450scc antibody at a dilution of 1∶200 overnight at 4°C. Biotin labeled secondary antibody was probed to slide sections for 1 hour at room temperature. The immune reaction complexes were detected using a streptavidin-HRP for 30 minutes and the resulting signal was developed with diaminobenzidine tablets (D-4293, Sigma). The sections were counterstained with Mayer’s Hematoxylin (S3309, DAKO) and mounted with glycerol (G-5516, Sigma) on glass slides.

Frozen ovaries were molded in OCT compound (Sakura Finetek, CA, USA), frozen to −20°C, sectioned at 10 um, and then stained with anti-CD31. Photomicrographs were taken with an Olympus microscope using Spot Imaging software version 2.1B (BX5ITF, Japan).

### Measurement of Serum Progesterone

Blood samples for the hormone assay were obtained at the time of euthanasia by cardiac puncture at 16 and 24 h after hCG injection. Sera were separated from whole blood and frozen until the time of analysis. Radioactive immunoassay (RIA) for plasma progesterone was performed following a standard procedure by the University of Virginia Center for Research in Reproduction Ligand Assay and Analysis Core of National Institute of Child Health and Human Development (Specialized Cooperative Centers Program in Reproductive Research) Grant U54-HD28934, University of Virginia, Charlottesville, VA. The sensitivity was 0.1 ng/ml, and the intra- and inter-assay variations for the RIA were each less than 10%.

### Statistical Analyses

Continuous data were initially analyzed with a Shapiro-Wilk test for normality. Statistical significance was assessed using a Fischer’s exact test for the presence of CL between unstimulated Edn2KO mice and heterozygote siblings. A student’s two-tailed *t-*test was used for ovarian weight and the number of oocytes retrieved from Edn2KO mice at hCG16. An ANOVA followed by Tukey’s post hoc was used for the number of oocytes retrieved from Edn2KO mice at hCG24. Serum progesterone levels in superovulated mice at hCG16, progesterone levels and oocytes ovulated in tezosentan treated mice, and oocytes ovulated in EDN2-supplemented mice were not normally distributed and were analyzed by a Mann-Whitney U test. Similarly, a Kruskal-Wallace test followed by Mann Whitney U tests were used to analyze serum progesterone across ovulated, non-ovulated, and control groups. *P*<0.05 was considered significant for all analyses used. Statistical analyses were performed with SPSS statistical software (SPSS, Inc., released 2009, PASW Statistics for Windows, Version 18.0, Chicago, IL).

## Results

### Edn2KO Mice are Overall Smaller in Body Size and have Lower Survival than Wild Type Littermates

Edn2KO mice were produced following the breeding scheme of Chang et?al. [41]. Edn2KO mice displayed shorter life spans and a smaller physique compared to control littermates as previously reported [27], [41]. Differences between littermate and Edn2KO mouse masses have been previously compared by Chang et?al. [33] and are consistent with our own (data not shown). Edn2KO female mice had lighter and shorter uteri and lighter ovaries (Fig. 1). Given their poor health, most of the following experiments were performed using immature female mice, except a few that survived up to the age of 7 weeks with thermal supplementation [41] that were used to assess adult ovaries following pubertal onset. Heterozygous littermates displayed no significant difference in the number of oocytes ovulated or in serum progesterone levels after induced ovulation compared to WT mice, nor any other morphological differences, and were used as controls for Edn2KO mice for the remainder of this study (Table 1).

**Table 1:** Table **1.** Ovarian function of heterozygous Edn2+/? mice and WT mice.

Assay time	Genotype	Numbers of ovulated oocytes/ovary		Genotype	Serum Progesterone ng/uL	
(h)	(n)	Average ± SEM	P-value	(n)	Average ± SEM	P-value
hCG 16	WT (8)	????±???		WT (7)	????±????	
	Edn2**^+/?^** (16)	????±???	0.940	Edn2**^+/?^** (4)	????±????	0.282

### Normal Folliculogenesis but no Corpora Lutea in the Post-pubertal Edn2KO Mouse Ovary

**Figure 2:**
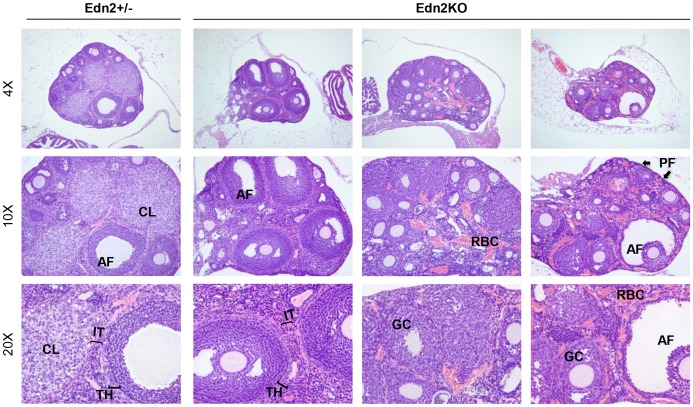
Ovarian histology of Edn2KO mice and heterozygous littermates. Ovaries collected from 5 to 7 week old Edn2KO mice and Edn2+/? littermates (n = 5 for each genotype) were serially sectioned and stained with hematoxylin and eosin. All ovaries contain follicles of various stages, but no CL were present in the Edn2KO ovaries. Representative images are displayed; the two Edn2KO ovaries at right display prominent congested blood vessels. CL: corpus luteum; AF: antral follicle; GC: granulosa cells; IT: interstitium; PF: primary follicles; TH: theca layer; RBC: red blood cells.

When examined histologically at the age of 5–7 weeks without any gonadotropin stimulation, follicular development in post-pubertal Edn2KO mice appeared qualitatively normal: preantral and antral follicles were present in the ovaries, though a varying degree of vascular congestion was seen in some Edn2KO ovaries (3/5) (Fig. 2). Red blood cells were visible in engorged vessels but did not appear to breach vascular walls and instead remained in discreet areas in an obvious branching pattern when viewed histologically. Granulosa cell and theca cell layers of the follicles and the interstitial layers of the ovary in these adult Edn2KO mice did not show any noticeable abnormalities, and no differences were apparent in the ovarian stroma between control and Edn2KO mice. Most obviously, no CL were present in any Edn2KO ovaries (0/5) whereas age-matching mouse ovaries had multiple CLs (5/5), p = 0.008 (Fig. 2).

### Edn2KO Ovaries Display Normal Vasculogenesis and Lack Excessive Atresia

**Figure 3:**
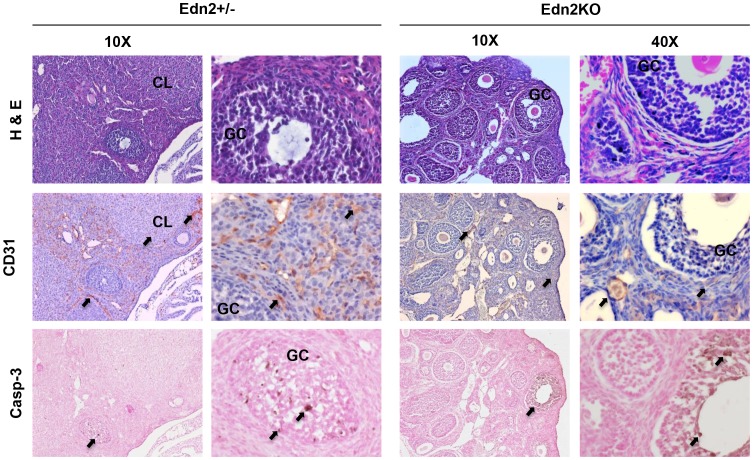
Vascularization and distribution of atretic follicles in the Edn2KO ovary. 25 day-old Edn2KO mice and heterozygous littermates were induced for superovulation. Ovaries were collected 16 hours after hCG injection and serially sectioned and stained with hematoxylin and eosin, or underwent immunohistochemical staining for CD31 (PECAM-1; an endothelial membrane marker) or Caspase 3 (Casp-3; an apoptotic cell marker). Dark brown membranal immunolabeling by anti-CD31 antibody presented multifocal staining in the medullary interstitium and the theca layer in both genotypes. Additional staining was present within the CL of Edn2+/? ovaries. Rare and multifocal anti-Casp-3 antibody immunolabeling was equally present in the cytoplasm of secondary and antral follicles of each genotype. There was no difference in endothelial cell or atretic staining observed between genotypes. Arrows indicate CD31 and Casp-3 staining; CL: corpus luteum; GC: granulosa cells.

To determine if defects seen in Edn2KO ovaries were due to increased atresia or poor vessel formation, 25 day-old mice were superovulated with hCG and sacrificed 16 hours later. Fixed ovaries (n = 4/group) were immunohistochemically stained for CD31 and Caspase-3 (Fig. 3). No differences were seen between control and Edn2KO ovaries for either staining pattern. CD31 staining for endothelial cells clearly marked large vessels in all ovaries, and also produced a fainter stain in the smaller vessels. Follicles were clearly demarcated with no intrafollicular staining. There was no difference in staining in the microvasculature between groups, except for positive staining in the young CL of WT ovaries which was absent in Edn2KO ovaries. Similarly, Caspase-3 staining for atresia showed no difference between groups. No obvious atresia was seen in all ovaries except rare positive Caspase-3 staining in the granulosa cells of specific secondary and antral follicles in each ovary (0–3/section), which possessed some cells with small, dark, picnotic nuclei visible with H&E staining (Fig. 3).

### Poor Ovulatory Response of Edn2KO Mice to Superovulation Induction

To further determine the ovulatory capacity of Edn2KO mouse ovaries, exogenous gonadotropins were injected into pre-pubertal mice. Due to difficulties generating and maintaining Edn2KO mice up to the ages to be used, this experimental set was performed four times over a period of 7 months with a small number of littermates each time. The first experiment was performed with two Edn2KO and four heterozygote mice. Superovulation was induced, and the oocyte number was counted at 16 hours after the hCG injection, as normal ovulation occurs between 12 to 14 hours after hCG injection in WT mice. Only 1 oocyte was retrieved from one of the two Edn2KO mice, whereas an average of 18.0±4.8 oocytes per ovary was released from the heterozygote controls (Table 2, top; p = 0.007). The superovulation experiments were continued 3 more times with two to three Edn2KO mice and matching numbers of heterozygote littermates each time, but the oocyte counting was performed at a later time point (24 hours after hCG injection) (Table 3). A later assessment time of the ovulatory response was done in consideration that oocyte release might be delayed in the Edn2KO mice. Consistent with the first experiment, the second and third experiments showed a severe ovulatory defect in the Edn2KO mice. Out of six Edn2KO mice examined, only two mice had 1 oocyte each in one of their oviducts. Littermates ovulated an average of 20.5±1.7 oocytes per ovary (Table 2, center; p<0.001). The fourth experiment was performed as above; unexpectedly, a normal range of oocytes (18.5±3.2/ovary) were retrieved from the Edn2KO mouse oviducts (Table 2, bottom; p = 0.604). Subsequent genotyping confirmed that these animals were indeed Edn2KO mice. When mice from the three experiments at hCG 24h were combined into Edn2KO and Edn2+/? groups, there was a significant difference in the number of oocytes ovulated (p<0.001). A significant difference remained when mice from all four experimental sets were combined together (p<0.001).

**Table 2:** Numbers of oocytes retrieved at hCG 16 hours.

Assay time	Genotype	Numbers of ovulated oocytes/ovary		
(h)	(n)	Average ± SEM	Range	P-value
hCG16	Edn2**^+/?^** (4)	18.0±4.8	3–36	
	Edn2KO (2)	0.3±0.3	0–1	0.007

**Table 3:** Numbers of oocytes retrieved at hCG 24 hours.

Assay time	Genotype	Numbers of ovulated oocytes/ovary		
(h)	(n)	Average ± SEM	Range	P-value
hCG 24	Edn2**^+/?^** (8)	20.5±1.7	10–32	*
	Edn2KO (6)	0.2±0.1	0–1	<0.001
	Edn2KO** (2)	18.5±3.2	10–24	0.604

*Edn2+/? mice were used for statistical comparison to either Edn2KO group. These Edn2KO** mice were from a single experimental trial.

### No Corpora Lutea in the Superovulation Induced Edn2KO Mouse Ovary

**Figure 4:**
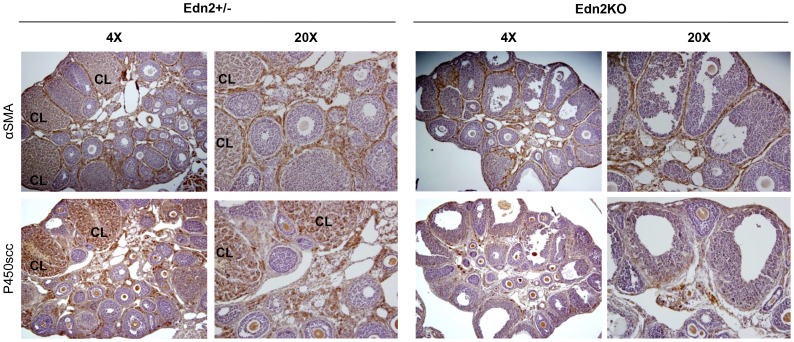
Lack of CL formation in the Edn2KO ovary following superovulation induction. Adjacent ovarian sections of the Edn2KO mice and heterozygous littermates collected at hCG24 were stained with αSMA antibody and P450scc antibody. Edn2+/? ovaries display αSMA and P450scc staining within CL, as well as in the interstitium around clearly demarcated follicles. In the Edn2KO ovaries, CL are absent and multiple large Graafian follicles are instead present in the periphery of the ovaries. Some, but not all, contain an oocyte with a partial or complete cumulus layer. Histology was similar between all Edn2KO ovaries regardless of number of oocytes ovulated. CL: corpus lutea.

**Figure 5:**
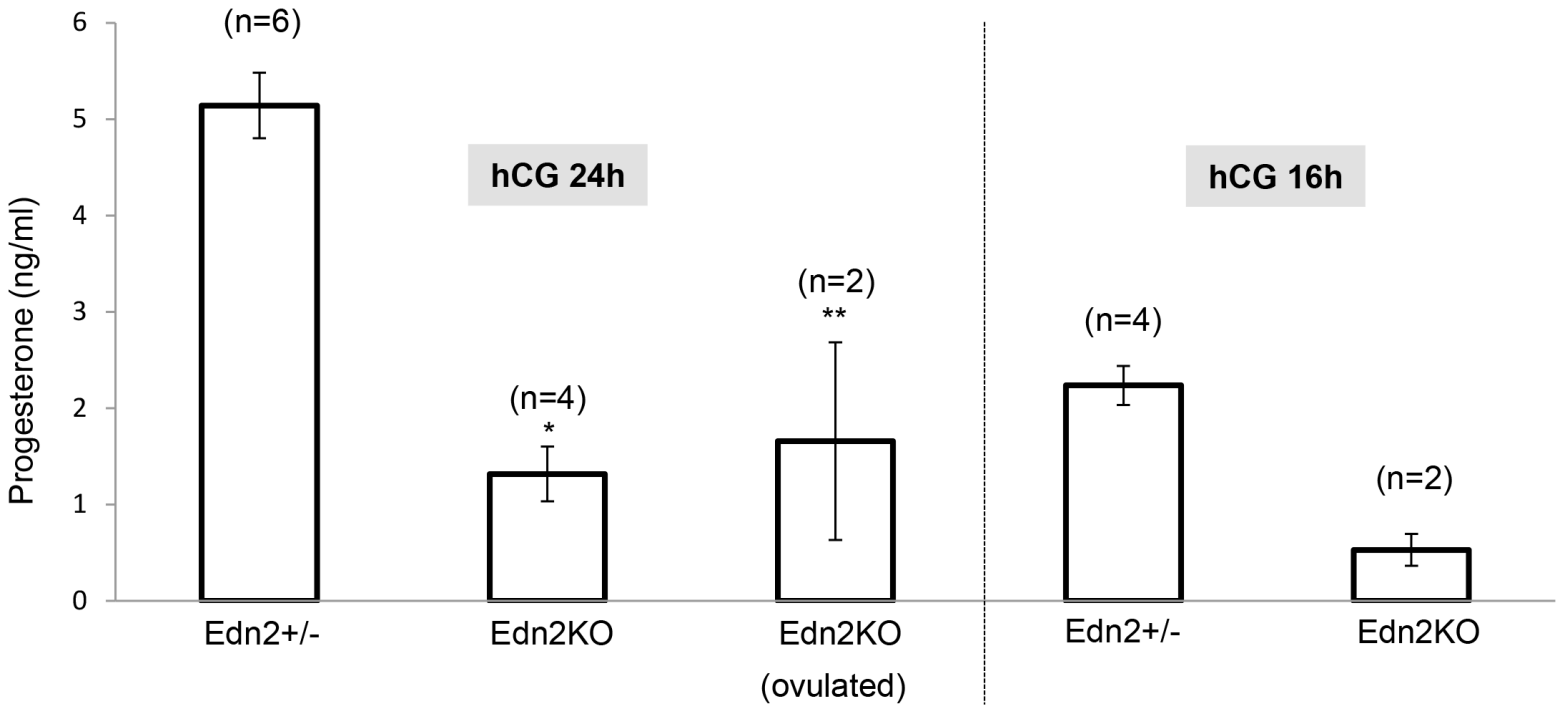
Serum progesterone concentrations. Blood samples were collected by cardiac puncture at 16h and 24h after hCG injection, and serum progesterone concentrations were measured by RIA. The numbers of mice (n) used for each group and the time points of assays are indicated. The progesterone concentrations in the Edn2KO mice that ovulated multiple oocytes at hCG 24 h were significantly lower than heterozygous littermates (p = 0.046), but similar to the Edn2KO mice that did not ovulate normally (p = 0.355). The mean serum progesterone concentrations were lower in Edn2KO mice than littermates when the concentrations were compared at hCG 16 h. Error bars (SEM) extend the range of recorded values for columns in which n = 2. For * and **, p = 0.011 and 0.046, respectively; for hCG 16h, p = 0.064.

The ovaries collected from the superovulation induction experiments were subjected to histological examination (Fig. 3–4), and progesterone concentration measurement was performed on serum samples (Fig. 5). Edn2KO mice that did not ovulate were devoid of CL. However, no CL were present even in the Edn2KO ovaries that did ovulate one or multiple oocytes (Fig. 4). Instead, multiple large structures closely resembling atretic Graafian follicles were present in these Edn2KO ovaries. These fluid-filled structures were lined by granulosa cells, some contained an oocyte with a partial or complete cumulus layer, and some possessed red blood cells; upon serial sectioning examination, not all structures contained an oocyte. Characteristic polyhedral luteal cells as expected in CL were not observed in Edn2KO ovaries. To mark both cells lining large vessels and those involved in microvasculature within the CL, ovaries were stained with anti-alpha smooth muscle actin (αSMA) antibody. In addition to large arteries and veins, theca externa layers around follicles and CL were clearly labeled in control ovaries, as well as microvessels within active CL, although staining was lighter than in large arteries. However, no staining was seen within follicles or other obvious demarcated boundaries of Edn2KO ovaries. This lack of staining confirms the inability of Edn2KO ovaries to form vascularized corpora lutea even when capable of releasing oocytes. Immunohistochemistry for cytochrome P450_scc_ expression was also performed to evaluate presence of a necessary steroidogenic enzyme for progesterone synthesis. Expression was absent in all follicles of the Edn2KO ovaries, though CL of heterozygous ovaries were darkly and poignantly stained (Fig. 4). Follicles that did not contain oocytes in Edn2KO mice did not stain positively for PF450_scc_, confirming lack of functionality (Fig. 4). Furthermore, significantly lower serum progesterone concentrations were present in Edn2KO mice compared to control mice at hCG 24h (Fig. 5). No difference was seen histologically in the theca-interstitial smooth muscle network between Edn2KO and control ovaries at either time point (Fig. 4).

### Ovulation and CL Formation in Edn2KO Mice Following Exogenous EDN2 Peptide Injection

**Figure 6:**
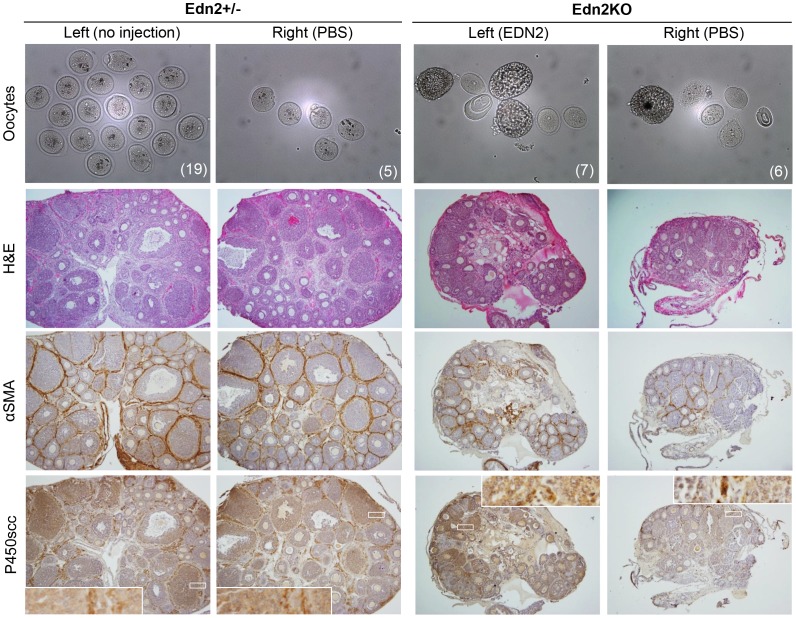
Ovulation and CL formation in Edn2KO mice after EDN2 peptide injection. Oocytes and ovaries were collected at hCG 16h from Edn2KO mice and control littermates that were induced for superovulation and were given intraovarian injections of EDN2 peptide, PBS, or no injection at hCG12. Ovarian sections were stained with H&E, or against αSMA or P450scc. Note that P450scc staining is present in the CL of Edn2+/? ovaries and in the granulosa cells of larger follicles of Edn2KO ovaries that had received EDN2 treatment, but not in vehicle-treated Edn2KO ovaries. The number of oocytes ovulated by each ovary is displayed in the corresponding oocyte image in the lower right corner. Histology images are taken at 4X; inset images of P450staining are at 40X.

We next sought to determine if direct EDN2 peptide administration to the ovary at the time of ovulation was sufficient to restore ovulation and CL formation. First, the feasibility of intraovarian injection was tested by comparing the number of oocytes ovulated upon super-ovulation induction between ovaries that were injected with PBS (right ovary) and those that were not injected (left; n = 5) in heterozygote mice. When examined at hCG 16h, slightly fewer, but a normal range of oocytes were found in the oviducts of PBS-injected ovaries compared to ovaries receiving no injection (Table 4, p = 0.028). We then compared the number of oocytes ovulated between PBS- and EDN2-injected ovaries in WT mice (n = 5). No significant difference was observed between treatments (p = 0.458). Lastly, Edn2KO mice (n = 3) similarly received either PBS or EDN2 injection into contralateral ovaries. All EDN2-injected ovaries ovulated (average 8.3 oocytes per ovary), whereas only one ovary ovulated among three PBS-injected ones did. The difference in oocytes ovulated in Edn2KO mice did not reach statistical significance (Table 4, p = 0.128), likely due to a small sample size and high variation in oocyte number in normal ovulation, though all EDN2-injected ovaries ovulated an increased number of oocytes relative to their contralateral PBS-injected counterparts. It was noted that small follicles were found among the oocytes released into the oviducts in both EDN2-inected and PBS-injected sides (Fig. 6).

**Table 4:** Numbers of oocytes retrieved after EDN2 injection.

Assay time	Genotype	Numbers of ovulated oocytes/ovary (respectively)		
(h)	(n)	PBS injected	Intact*/EDN2 injected	P-value
hCG 16	Edn2^+/?^ (5)*	19, 9, 19, 5, 15	26, 20, 20, 19, 23	0.028*
	WT (5)	48, 21, 18, 12, 21	42, 17, 19, 17, 17	0.458
	Edn2KO (3)	0, 6, 0	2, 7, 16	0.127

*Edn2+/? mice (5) were given no injection in this experiment and oocyte numbers are provided respective to ovaries from the same animal. WT (5) and Edn2KO (3) mice received either EDN2 or PBS into each ovary of the same animal.

**Figure 7:**
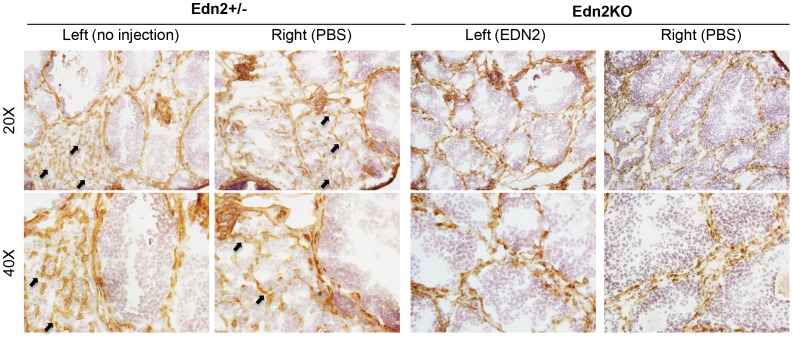
CD31 expression in Edn2KO and Edn2+/? ovaries after EDN2, vehicle, or no injection. Frozen sections were made from Edn2+/? or Edn2KO mouse ovaries collected at hCG16. Mice received injection with EDN2 peptide, PBS, or no injection at hCG12. Ovaries were stained with CD31 (PECAM-1) to visualize the vascular network. Note that positive staining was seen inside the CL of heterozygote ovaries, but was not present inside any demarcated structures of Edn2KO ovaries treated with EDN2 or PBS. Arrows indicate vascularization within representative CL of ovaries treated with PBS or no injection.

Upon ovarian histology, no CL were observed in vehicle treated Edn2KO ovaries, though CL were observed in all heterozygote ovaries. EDN2-treated Edn2KO ovaries had increased staining for P450_scc_ staining within structures that appeared as small CL or large follicles, though this was absent in PBS-treated Edn2KO ovaries (Fig. 6). Darker staining was seen in CL of PBS-treated or un-injected control ovaries. No significant differences in αSMA staining were noted in all treatments. When frozen ovarian sections of mice from the same treatment were stained for CD31, microvessel expression was obvious in the CL of all heterozygote animals. However, no vascularization was visible in the Edn2KO ovaries of either EDN2 or PBS injection treatments (Fig. 7).

### Endothelin Receptor Antagonism Decreases Ovulation Rate, Progesterone Synthesis, and CL Vascularization

**Figure 8:**
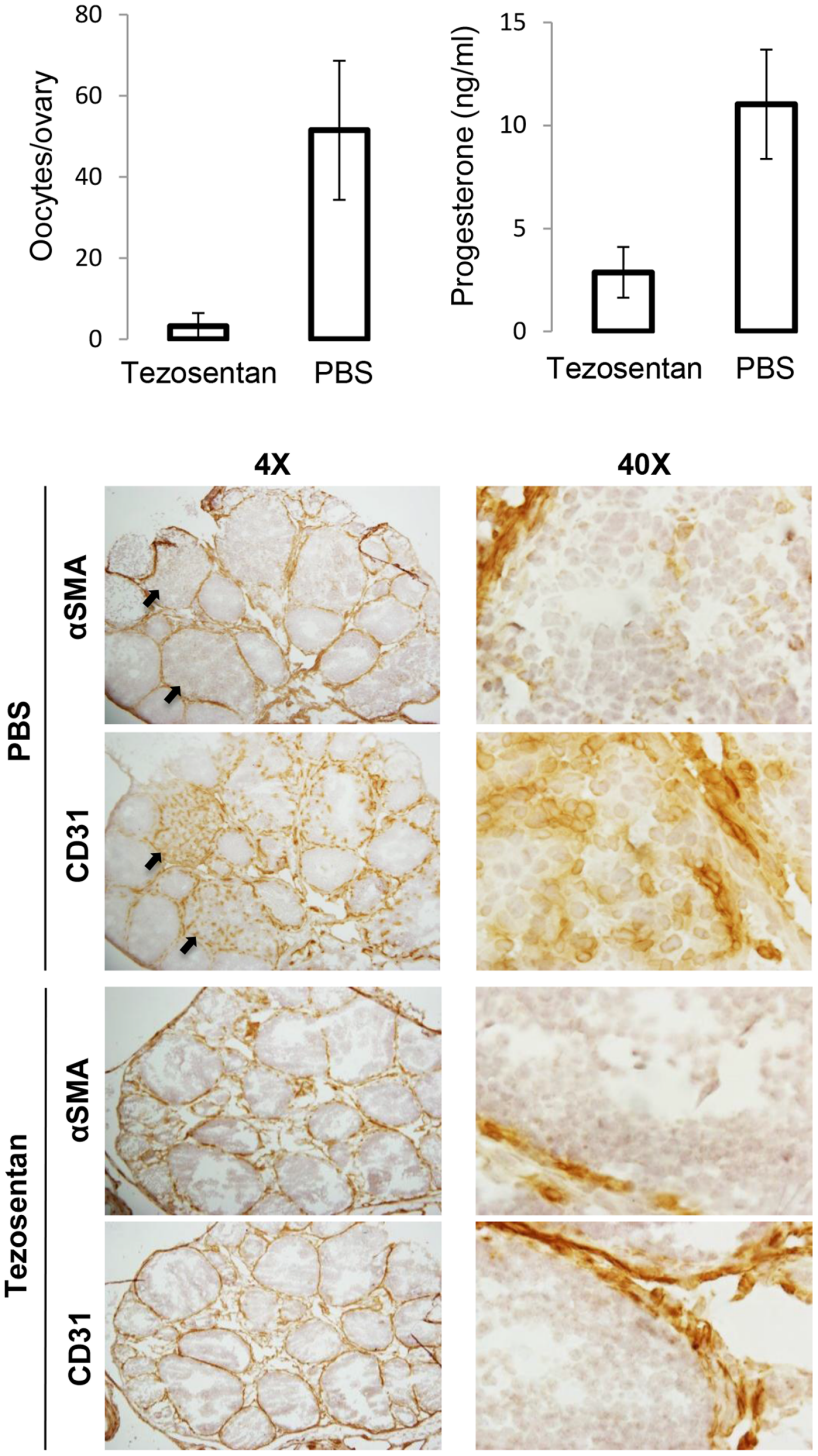
Inhibition of endothelin receptors prevents CL formation and progesterone synthesis. WT mice were induced for superovulation and received vehicle or tezosentan injections every 2 hours from hCG12 to hCG22. Mice (n = 4) were sacrificed at hCG24 hours. Tezosentan-treated mice ovulated significantly fewer oocytes (p = 0.038) and had lower serum progesterone levels (p = 0.043). Frozen ovarian sections were stained for CD31 and αSMA. Note that CL from PBS-injected mice showed clear CD31 staining in the CL, but CD31 staining was limited to the theca layers of tezosentan-injected ovaries. Little αSMA staining was seen in the CL of PBS treated mice or within tezosentan treated mouse ovaries. Arrows indicate CL in PBS-treated ovaries.

To determine if endothelin receptors are necessary for CL formation, tezosentan (a dual endothelin receptor inhibitor) was injected to superovulation-induced immature WT mice every 2 hours from hCG 12h to hCG 22h. Ovulation and CL formation normally begin at hCG 12h, and we intended to antagonize endothelin receptors during the period of CL formation following ovulation by beginning tezosentan injections at the presumed time of ovulation. Though fewer oocytes (3.3±3.3/ovary) were ovulated in tezosentan-injected mice than in vehicle treated mice (51.5±17.1/ovary; p = 0.038), ovulation did occur, as expected. Immunohistochemistry of frozen ovarian sections showed light αSMA staining in the young CL of vehicle treated ovaries, but no such αSMA-positive structure similar to a CL was found in the ovaries of tezosentan treated mice. CD31 staining was poignantly visible in the small vessels of the CL of vehicle treated animals, but was absent in tezosentan-treated mouse ovaries (Fig. 8). Consistent with lack of CL formation, the tezosentan treated mice had significantly lower serum progesterone levels (2.9±1.2ng/ml) than vehicle treated mice (11.0±2.7ng/ml; p = 0.043; Fig. 8).

## Discussion and Conclusions

*Edn2* was previously identified as an inducible gene that is expressed in granulosa cells immediately prior to ovulation for a short duration [22]. Here we hypothesized that EDN2 triggers oocyte expulsion and is concurrently necessary for CL formation. We examined these events using both Edn2KO and WT mice. These mice consistently displayed impaired ovulation and a lack of CL formation: in post-pubertal mice that were not stimulated by gonadotropins, ovaries were devoid of CL in contrast to multiple CL in age-matching heterozygous littermates. This is suggestive of an expected inherent defect in ovulation, given previous data on ovulation in Endothelin receptor antagonized mice [11], [13], [14]. Pre-pubertal Edn2KO mice were tested using superovulation protocol to quantify ovulatory defects and demonstrate that the effect of EDN2 loss was intrinsic to the ovary. The most striking finding from this study is the critical role EDN2 has in the formation of the CL. Pre-pubertal Edn2KO mice undergoing superovulation generally fail to ovulate and in no instances were CL formed. The absence of CL but presence of antral follicles in the Edn2KO mouse ovary is an indication that lack of CL was not due to abnormal folliculogenesis. Multiple atretic Graafian follicles seen in Edn2KO mice were absent in control ovaries, indicating that the ovulated follicles failed to transform into CL, despite the exogenous gonadotropin surge. In agreement, progesterone levels were also significantly lower in all Edn2KO mice than control mice. To rule out another systemic defect resulting from global EDN2 loss, Endothelin receptors were pharmacologically antagonized in WT mice and similar results were obtained: treated mice ovulated few oocytes, had low progesterone levels, and failed to form identifiable CL. Finally, replacement of EDN2 peptide into several Edn2KO mouse ovaries suggested an augmented ovulation and increased steroidogenic enzyme expression within the ovary, though significance was not reached.

We were particularly intrigued by the lack of CL in the ovaries of the two outlying Edn2KO mice that did ovulate a normal range of oocytes. Incorrect genotyping was ruled out and, in consideration of the established ability of EDN2 to induce contraction in ovarian tissues [13], [16], it is speculated that contraction might be induced in these Edn2KO ovaries by some molecules(s) such as prostaglandins that are produced in the ovary [42], [43], triggering oocyte expulsion. Both of these Edn2KO mice had low serum progesterone concentration, strengthening the assumption that EDN2 is necessary for CL formation even when oocyte release occurs. We conclude by noting that the Edn2KO ovary is defective in ovulation and progesterone synthesis and, consequently, EDN2 expression in the ovary is necessary for pregnancy establishment and maintenance as well as normal ovulation.

This novel role of EDN2 as a critical factor in luteal formation is in contrast to the well-established functional role of EDN1 as a luteolytic factor, though both molecules have equivalent affinities for each receptor in the endothelin system. Ovarian EDN1 is one of many locally produced agents that regulate luteal function [44]–[49]. Luteal EDN1 concentration rises soon after the increase of uterine PGF2α secretion [50]–[52], and EDN1 inhibits gonadotropin-stimulated progesterone synthesis in luteal cells and promotes luteolysis [53]. The findings from this study are particularly relevant as Klipper and colleagues suggested an important role for EDN2 in CL formation in bovines [17]. Using bovine luteinizing granulosa cells, EDN2 induced the expression of vascular endothelial growth factor (VEGF), an angiogenic factor and critical player in CL formation. Therefore, it may be of interest to determine if VEGF expression is affected in the granulosa cells of Edn2KO mice. Rattner et?al. have also recently shown that EDN2 promotes endothelial cells to maintain a tip-cell state during angiogenesis, and that EDN2 has the ability to ‘override’ VEGF signaling [54]. Other reports indicate that EDN2 may act as a macrophage chemoattractant [32], [34], which may induce or modify the activity of matrix metalloproteinases (MMPS) or tissue inhibitors of MMPS (TIMPs) [55]–[57]. MMP activity has been shown to be necessary for endothelial cell release during angiogenesis [58]. Based on those previous findings, we summarily speculate that EDN2 concurrently increases smooth muscle tension, augments or induces VEGF secretion, and increases MMP activity through modified secretion or macrophage recruitment, collectively triggering ovulation and luteal angiogenesis for CL formation.

We acknowledge that Edn2KO mice are weak and this exists as a confounding factor in possible conclusions drawn from usage of that model. Such health conditions can compromise follicle development and ovarian function in general. However, the presence of non-atretic follicles of all stages in Edn2KO mice, the capability of some Edn2KO mice to release oocytes after superovulation induction and yet fail to form CL, and, most firmly, the equivalent results in WT mice using pharmacological inhibition all strongly suggest the observed lack of CL is derived from loss of EDN2 in ovary. An ovary-specific or granulosa-cell specific Edn2KO animal model is needed to solidify this role for EDN2 in female reproduction and assuage concerns from systemic health issues and the low survival rate of Edn2KO mice past weaning. Forthcoming exploration may lead to a downstream target of *Edn2* that may be used as a novel non-hormonal contraceptive target or as a messenger to increase fertility by increasing ovulatory capacity and CL formation. Future work may additionally examine other post-developmental organ-specific roles for EDN2 in vascular formation.
